# Fauna Europaea: Hymenoptera – Apocrita (excl. Ichneumonoidea)

**DOI:** 10.3897/BDJ.3.e4186

**Published:** 2015-03-20

**Authors:** Mircea-Dan Mitroiu, John Noyes, Aleksandar Cetkovic, Guido Nonveiller, Alexander Radchenko, Andrew Polaszek, Fredrick Ronquist, Mattias Forshage, Guido Pagliano, Josef Gusenleitner, Mario Boni Bartalucci, Massimo Olmi, Lucian Fusu, Michael Madl, Norman F Johnson, Petr Jansta, Raymond Wahis, Villu Soon, Paolo Rosa, Till Osten, Yvan Barbier, Yde de Jong

**Affiliations:** ‡ Alexandru Ioan Cuza University, Faculty of Biology, Iasi, Romania; § Natural History Museum, London, United Kingdom; | University of Belgrade, Faculty of Biology, Belgrade, Serbia; ¶ Nusiceva 2a, Belgrade (Zemun), Serbia; # Schmalhausen Institute of Zoology, Kiev, Ukraine; ¤ Uppsala University, Evolutionary Biology Centre, Uppsala, Sweden; « Swedish Museum of Natural History, Stockholm, Sweden; » Museo Regionale di Scienze Naturi, Torino, Italy; ˄ Private, Linz, Austria; ˅ Museo de “La Specola”, Firenze, Italy; ¦ Università degli Studi della Tuscia, Viterbo, Italy; ˀ Alexandru Ioan Cuza University of Iasi, Faculty of Biology, Iasi, Romania; ˁ Naturhistorisches Museum Wien, Wien, Austria; ₵ Museum of Biological Diversity, Columbus, OH, United States of America; ℓ Charles University, Faculty of Sciences, Prague, Czech Republic; ₰ Gembloux Agro bio tech, Université de Liège, Gembloux, Belgium; ₱ University of Tartu, Institute of Ecology and Earth Sciences, Tartu, Estonia; ₳ Via Belvedere 8d, Bernareggio, Italy; ₴ Private, Murr, Germany; ₣ Université de Mons-Hainaut, Mons, Belgium; ₮ University of Amsterdam - Faculty of Science, Amsterdam, Netherlands; ₦ University of Eastern Finland, Joensuu, Finland

**Keywords:** Biodiversity informatics, Hymenoptera, Apocrita, Fauna Europaea, taxonomic indexing

## Abstract

*Fauna Europaea* provides a public web-service with an index of scientific names (including important synonyms) of all living European land and freshwater animals, their geographical distribution at country level (up to the Urals, excluding the Caucasus region), and some additional information. The *Fauna Europaea* project covers about 230,000 taxonomic names, including 130,000 accepted species and 14,000 accepted subspecies. This represents a huge effort by more than 400 contributing specialists throughout Europe and is a unique (standard) reference suitable for many users in science, government, industry, nature conservation and education.

Hymenoptera is one of the four largest orders of insects, with about 130,000 described species. In the *Fauna Europaea* database, ‘Hymenoptera - Apocrita (excluding Ichneumonoidea)’ comprises 13 superfamilies, 52 families, 91 subfamilies, 38 tribes and 13,211 species. The paper includes a complete list of taxa dealt with, the number of species in each and the name of the specialist responsible for data acquisition. As a general conclusion about the European fauna of Hymenoptera, the best known countries in terms of recorded species are those from northwestern Europe, with the least known fauna probably in the more eastern and southeastern parts of Europe.

## Introduction

The European Commission published the European Community Biodiversity Strategy, providing a framework for development of Community policies and instruments in order to comply with the Convention on Biological Diversity. The Strategy recognises the current incomplete state of knowledge at all levels concerning biodiversity, which is a constraint on the successful implementation of the Convention. *Fauna Europaea* contributes to this Strategy by supporting one of the main themes: to identify and catalogue the components of European biodiversity into a database to serve as a basic tool for science and conservation policies.

In regard to biodiversity in Europe, science and policies depend on knowledge of its components. The assessment of biodiversity, monitoring changes, sustainable exploitation of biodiversity, and much legislative work depends upon a validated overview of taxonomic biodiversity, in which *Fauna Europaea* plays a major role, providing a web-based information infrastructure with an index of scientific names (including important synonyms) of all living European land and freshwater animals, their geographical distribution at country level and some additional optional information. In this sense the *Fauna Europaea* database provides a unique reference for many user-groups such as scientists, governments, industries, conservation communities and educational programs.

*Fauna Europaea* kicked-off in 2000 as an EC-FP5 four years project, delivering its first release in 2004 (Jong et al. 2014). After thirteen years of steady progress, in order to efficiently disseminate the Fauna Europaea results and to increase the acknowledgement of the Fauna Europaea contributors, novel e-Publishing tools have been applied to prepare data-papers of all major taxonomic groups. For this purpose a special Biodiversity Data Journal Series has been compiled, called Contributions on Fauna Europaea. This work was initiated during the ViBRANT project and is further supported by the recently started EU BON project. This paper holds the first publication of the Fauna Europaea Hymenoptera - Apocrita (excluding Ichneumonoidea) data sector as a BDJ data paper.

Within the EU BON project further steps will be made to implement *Fauna Europaea* as a basic tool and standard reference for biodiversity research and to evaluate taxonomic expertise capacity in Europe. The *Fauna Europaea* data-papers will contribute to a quality assessement on biodiversity data by providing estimates on gaps in taxonomic information and knowledge.

## General description

### Purpose

*Fauna Europaea* is a database of the scientific names and distribution of all extant, currently known multicellular European land and freshwater animal species assembled by a large network of experts. An extended description of the Fauna Europaea project can be found in Jong et al. 2014. A summary is given in the sections below.

The Hymenoptera - Apocrita (excluding Ichneumonoidea) is one of the 58 *Fauna Europaea* major taxonomic groups, covering 13,211 species (Fig. 2) and represented by a network of 20 specialists (Table 1). The current data-paper respects the organization of the animal groups present in the *Fauna Europaea* database.

### Additional information

Hymenoptera is one of the four largest orders of insects (beside Coleoptera, Diptera and Lepidoptera), with about 130,000 described species. Their success is probably due to the tremendous range of ecological and behavioral adaptations. Two main groups (usually treated as suborders) are generally recognized within Hymenoptera: the paraphyletic Symphyta (sawflies and horntails) and the monophyletic Apocrita (bees, ants and wasps). Traditionally, Apocrita is divided in the paraphyletic Parasitica (the ovipositor retains its primitive role in egg-laying) and the monophyletic Aculeata (the ovipositor is modified for stinging) (Vilhelmsen 2001, Schulmeister et al. 2002, Heraty et al. 2011).

The ecology and biology of the species from the above families are extremely diverse. In their larval stage most species are carnivorous, feeding mainly on other insects or spiders, but some groups are specialized on other diets such as nectar and pollen (e.g. Apidae), vegetable tissues (e.g. Cynipidae), or are omnivorous (e.g. Formicidae). Among the carnivorous species, most are parasitoids i.e. the free-living adult usually searches a host (the egg, larva, pupa, or even the adult of mostly another insect) and its larva (solitary parasitoid) or larvae (gregarious parasitoid) will then develop inside (endoparasitoid) or outside (ectoparasitoid) that host, almost invariably killing it.

The group contains many species of parasitoids frequently used in biological control e.g. *Trichogramma* spp. (Chalcidoidea: Trichogrammatidae), but also the smallest known insect, the wingless male of *Dicopomorpha echmepterygis* Mockford (Chalcidoidea: Mymaridae), an egg parasitoid of only 0.13 mm in length, and the smallest winged insect, some females of *Kikiki huna* Huber & Beardsley (Chalcidoidea: Mymaridae) being only 0.16 mm in length. Members of Apoidea are among the most important pollinator agents in ecosystems containing flowering plants. A few species are regarded as pests (some sawflies, ants, and wasps).

In the *Fauna Europaea* database, Hymenoptera - Apocrita (excluding Ichneumonoidea) comprises 13 superfamilies, 54 families and 91 subfamilies (see taxonomic coverage). Some recent changes in the classification of Hymenoptera Apocrita will be included in the next version, such as the treatment of Scelionidae as a subfamily of Platygastridae (Scelioninae) (Sharkey 2007​), the inclusion of *Cratomus* Dalman and *Panstenon* Walker (Pteromalidae: Cratominae, Panstenoninae) in Pteromalinae, the inclusion of Epichrysomallinae in Pteromalidae (Heraty et al. 2013), etc. A different classification system for Apoidea (such as the one in Checklist of the Western Palaearctic Bees) will also be considered following a consensus decision of bee specialists.

Fig. 4

**Gap estimates in Fauna Europaea:** Despite recent progress, it is important to note that we still know very little of the fauna of Hymenoptera for Europe. It is almost certain (if we use the UK fauna - by far the best known in Europe - as a guide and extrapolate from there) that the order Hymenoptera, in terms of species richness, would be far greater than that for Coleoptera. Currently the British Isles fauna of Hymenoptera stands at 7761 species, being the largest insect order in the region, ahead of Coleoptera and Diptera (Broad 2014).

Estimated gaps in terms of described species that are known from Europe, but are not currently included in the database are presented in Table 1. They range from zero for many families up to about 5-10% for other groups, and are expected to be filled in the next version of the database. Country gaps are not included in this analysis, but are expected to be higher in south-eastern European countries, where studies of Hymenoptera Apocrita fauna are still scarce compared with the north-western countries. The best known countries in Europe are probably UK (<80%), Sweden (<50%), ex Czechoslovak Republic (<50%), Germany (<50%), Italy (<50%), France (<30%), and Spain (<30%), with the least known fauna probably in the more eastern and southeastern parts of Europe such as Romania, Bulgaria, or Greece (probably all <20%) (Noyes, unpublished data).

With regard to the undescribed taxa, it would be generally highly speculative to estimate the potential number of new species for most families, especially for highly diverse groups containing minute species, such as Chalcidoidea, where possibly hundreds of new species await discovery. For other better studied groups such as Chrysididae, it is estimated that a large number of subspecies could be errected to species level, thus increasing the total number of valid taxa with about 50 species. In other groups it is also possible that the number of new synonyms will proove to be approximately equal to the number of newly described taxa, so that the total number of taxa will not become significantly higher.

Fig. 5

In addition, the number of taxonomists is continuously decreasing: unfortunatelly some excellent specialists are either deceased (Dr Till Osten and Dr Guido Nonveiller) or are retired and inactive (Table 2). If young taxonomists will not fill up these gaps, we will eventually end up not being able to identify most of the European biodiversity.

## Project description

### Title

This BDJ data paper includes the taxonomic indexing efforts in *Fauna Europaea* on European Hymenoptera - Apocrita covering the first two versions of Fauna Europaea worked on between 2000 and 2013 (up to version 2.6).

### Personnel

The taxonomic framework of Fauna Europaea includes partner institutes providing taxonomic expertise and information, and expert networks managing data collation.

Every taxonomic group is covered by at least one Group Coordinator responsible for the supervision and integrated input of taxonomic and distributional data of a particular group. For Hymenoptera - Apocrita the responsible Group Coordinators were John Noyes (version 1) and Mircea-Dan Mitroiu (version 2).

The *Fauna Europaea* checklist would not have reached its current level of completion without the input from several groups of specialists. The formal responsibility of collating and delivering the data of relevant families has resided with the Taxonomic Specialists (see Table 1), while Associate Specialists deserve credit for their important contributions at various levels, including particular geographic regions or (across) taxonomic groups.

Data management tasks are carried out by the *Fauna Europaea* project bureau. During the project phase (until 2004) a network of principal partners took responsability for various management tasks: Zoological Museum Amsterdam (general management & system development), Zoological Museum of Copenhagen (data collation), National Museum of Natural History in Paris (data validation) and Museum and Institute of Zoology in Warsaw (NAS extension). Once the formal end of the project ended (2004-2013) all tasks were were taken over by the Zoological Museum Amsterdam.

### Study area description

The study area covers the European mainland (Western Palaearctic), including the Macaronesian islands, excluding the Caucasus, Turkey, Arabian Peninsula and Northern Africa.

### Design description

Standards. Group coordinators and taxonomic specialists have to deliver the (sub)species names according to strict standards. The names provided by FaEu are scientific names. The taxonomic scope includes issues like (1) the definition of criteria used to identify the accepted species-group taxa, (2) the hierarchy (classification scheme) for the accommodation of the all accepted species, (3) relevant synonyms, and (4) the correct nomenclature. The *Fauna Europaea* 'Guidelines for Group Coordinators and Taxonomic Specialists', include the standards, protocols, scope, and limits that provide the instructions for all of the more than 400 specialists contributing to the project.

**Figure 1.**
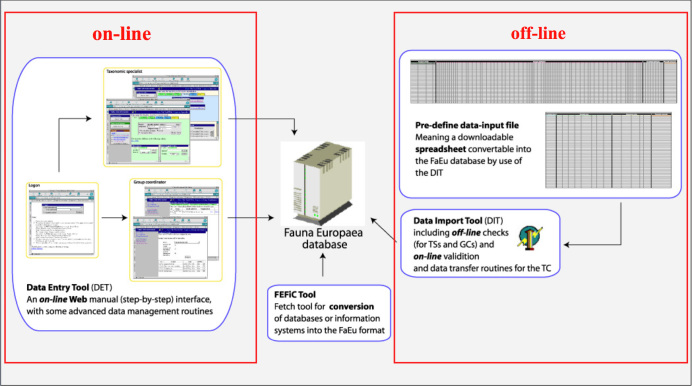
Fauna Europaea on-line (browser interfaces) and off-line (spreadsheets) data entry tools.

Data management. The data could either be entered offline into a preformatted MS-Excel worksheet or directly into the *Fauna Europaea* transaction database using an online browser interface (see Fig. 1). Since 2013 the data servers are hosted at the Museum für Naturkunde in Berlin (migrated from ZMA-UvA).

Data set. The *Fauna Europaea* basic data set consists of: accepted (sub)species names (including authorship), synonymous names (including authorship), taxonomic hierarchy / classification, misapplied names (including misspellings and alternative taxonomic views), homonym annotations, expert details, European distribution (at country level), Global distribution, taxonomic reference (optional), occurrence reference (optional).

### Funding

*Fauna Europaea* was funded by the European Commission under the Fifth Framework Programme and contributed to the Support for Research Infrastructures work programme with Thematic Priority Biodiversity (EVR1-1999-20001) for a period of four years (1 March 2000 - 1 March 2004), including a short 'NAS extension', allowing EU candidate accession countries to participate. Follow-up support was given by the EC-FP5 EuroCAT project (EVR1-CT-2002-20011), by the EC-FP6 ENBI project (EVK2-CT-2002-20020), by the EC-FP6 EDIT project (GCE 018340), by the EC-FP7 PESI project (RI-223806) and by the EC-FP7 ViBRANT project (RI-261532). Continuing management and hosting of the Fauna Europaea services was supported by the University of Amsterdam (Zoological Museum Amsterdam) and SARA/Vancis. Recently the hosting of Fauna Europaea was taken over by the Museum für Naturkunde in Berlin, supported by the EC-FP7 EU BON project (grant agreement №308454).

For preparing the Hymenoptera - Apocrita (excluding Ichneumonoidea) data-paper additional support was received from a grant of the Romanian National Authority for Scientific Research, CNCS–UEFISCDI, project number PN–II–RU–TE–2012–3–0057 to MDM.

## Sampling methods

### Study extent

See spatial coverage and geographic coverage descriptions.

### Sampling description

*Fauna Europaea* data have been assembled by principal taxonomic experts, based on their individual expertise, including literature study, collection research, and field observations. In total no fewer than 476 experts contributed taxonomic and/or faunistic information for *Fauna Europaea*. The vast majority of these experts are from Europe (including EU non-member states). As a unique feature, *Fauna Europaea* funds were set aside for paying/compensating for the work of taxonomic specialists and group coordinators (around five Euro per species).

To facilitate data transfer and data import, sophisticated on-line (web interfaces) and off-line (spreadsheets) data-entry routines have been built, well integrated within an underlying central *Fauna Europaea* transaction database (see Fig. 1). This includes advanced batch data import routines and utilities to display and monitor the data processing within the system. In retrospect, it seems that the off-line submission of data was probably the best for bulk import during the project phase, while the on-line tool was preferred to enter modifications in later versions. This system worked well until its replacement in 2013.

The *Fauna Europaea* index via the web-portal was firstly released on 27^th^ September 2004, the most recent release (version 2.6.2) was launched on 29th August 2013. An overview of *Fauna Europaea* releases can be found here: http://www.faunaeur.org/about_fauna_versions.php.

Fig. 6

### Quality control

*Fauna Europaea* data are unique in the sense that they are fully expert-based. Selecting leading experts for all groups included a principal assurance of the systematic reliability and consistency of the *Fauna Europaea* data.

Further, all *Fauna Europaea* data sets are intensively reviewed at regional and thematic validation meetings, at review sessions on taxonomic symposia (for some groups), by *Fauna Europaea* Focal Points (during the FaEu-NAS and PESI projects) and by various end-users sending annotations using the web form on the web-portal. Additional validation on gaps and correct spelling was effected at the validation office in Paris.

Checks on technical and logical correctness of the data have been implemented in the data entry tools, including around 50 "Taxonomic Integrity Rules". This validation tool proved to be of huge value for both the experts and project management, and significantly contribute(d) to preparation of a remarkably clean and consistent data set. This thorough reviewing makes *Fauna Europaea* the most scrutinised data sets in its domain.

In conclusion (see above), recognised gaps in Hymeneoptera - Apocrita (excluding Ichneumonoidea) include: slow up-dating of data-sets (with both faunistic and taxonomic information) for several groups e.g. Apidae or Cynipoidea; and few faunistic data for some groups e.g. Chalcidoidea, Platygastroidea or Proctotrupoidea, especially in south-eastern European countries.

To optimise the use and implementation of a uniform and correct nomenclature, also following the global efforts on establishing a so-called 'Global Names Architecture' (Pyle and Michel 2008, Patterson et al. 2010), a cross-referencing of the *Fauna Europaea* Hymenoptera - Apocrita (excluding Ichneumonoidea) data-set with relevant nomenclators, including the Universal Chalcidoidea Database, is recommended as well as a connection with relevant name services (like Hymenoptera Name Server). In addition, a interlinkage with relevant Hymenoptera information services (like Hymenoptera Online, Atlas Hymenoptera, BWARS and HymIS), regional data portals (like Forum Entomologi Italiani and eBiodiversity) and databases dedicated to smaller groups (like Chrysis.net, Bombus, Palaearctic Osmiine Bees and Checklist of the Western Palaearctic Bees) is proposed.

Fig. 7

### Step description

By evaluating team structure and life cycle procedures (data-entry, validation, updating, etc.), clear definitions of roles of users and user-groups, according to the taxonomic framework were established, including ownership and read and write privileges, and their changes during the project life-cycle. In addition, guidelines on common data exchange formats and codes have been issued (see also the 'Guidelines' document).

## Geographic coverage

### Description

Species and subspecies distributions in *Fauna Europaea* are registered at least at country level, defined politically. For this purpose the FaEu geographical system basically follows the TDWG 2.0 standards. The covered area includes the European mainland (Western Palaearctic), plus the Macaronesian islands (excl. Cape Verde Islands), Cyprus, Franz Josef Land and Novaya Zemlya. Western Kazakhstan and the Caucasus are excluded (see Fig. 3).

The focus is on species (or subspecies) of European multicellular animals of terrestrial and freshwater environments. Species in brackish waters, occupying the marine/freshwater or marine/terrestrial transition zones, are generally excluded.

### Coordinates

Mediterranean (N 35°) and Arctic Islands (N 82°) Latitude; Atlantic Ocean (Mid-Atlantic Ridge) (W 30°) and Urals (E 60°) Longitude.

## Taxonomic coverage

### Description

The *Fauna Europaea* database contains the scientific names of all living European land and freshwater animal species, including numerous infra-groups and synonyms. More details about the conceptual background of *Fauna Europaea* and standards followed are described in the project description papers (Jong et al. 2014).

**Figure 2.**
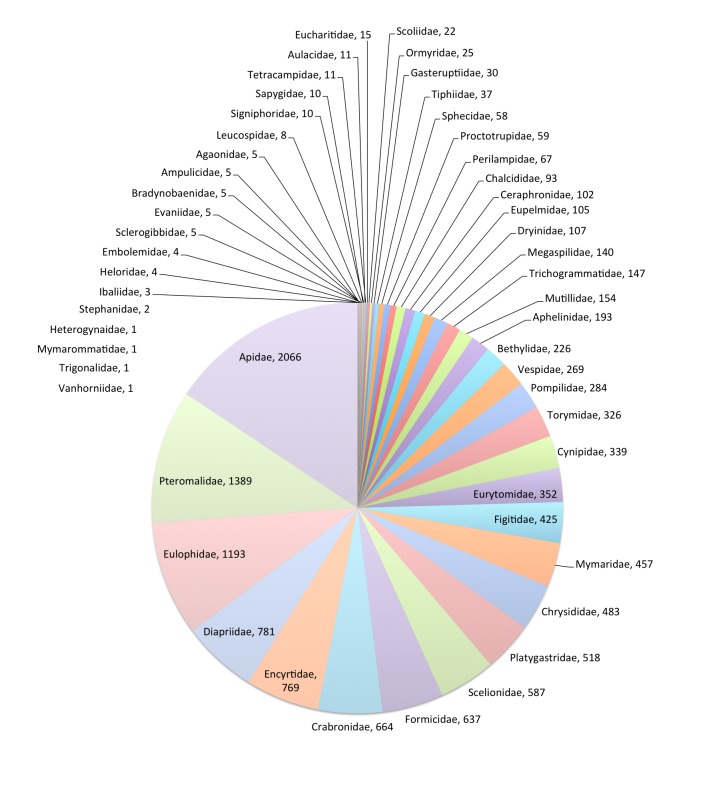
FaEu Hymenoptera-Apocrita (excluding Ichneumonoidea) species per family. See Table 1 for family statistics.

**Figure 3.**
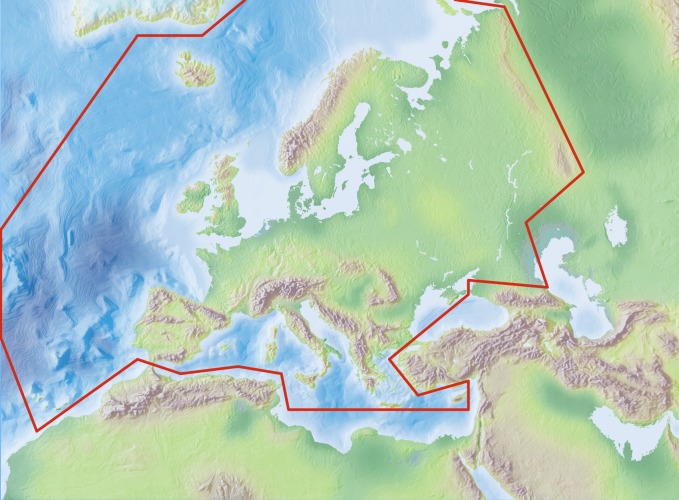
Fauna Europaea geographic coverage ('minimal Europe').

This data paper covers the Hymenoptera - Apocrita (excluding Ichneumonoidea) content of Fauna Europaea, including 52 families, 13,211 species, 826 subspecies and 5,676 (sub)species synonyms (see Fig. 2). Higher ranks are given below, the species list can be downloaded from the Fauna Europaea portal (see: Data resources).

The classification used in the *Fauna Europaea* database and consequently in this data-paper follows the opinions of the experts listed above. Readers should be aware that other classifications may exist. For example, regarding the Apidae, some specialists prefer to use several families instead of one (i.e. Andrenidae, Apidae, Colletidae, Halictidae, Megachilidae and Melittidae) (e.g. Patiny et al. 2009).

### Taxa included

**Table:** 

Rank	Scientific Name	Common Name
kingdom	Animalia	
subkingdom	Eumetazoa	
phylum	Arthropoda	
subphylum	Hexapoda	
class	Insecta	
order	Hymenoptera	
suborder	Apocrita	
superfamily	Apoidea	
family	Ampulicidae	
tribe	Ampulicini	
tribe	Dolichurini	
family	Apidae	
family	Crabronidae	
subfamily	Astatinae	
subfamily	Bembicinae	
tribe	Alyssontini	
tribe	Bembicini	
tribe	Nyssonini	
subfamily	Crabroninae	
tribe	Crabronini	
tribe	Larrini	
tribe	Miscophini	
tribe	Oxybelini	
tribe	Palarini	
tribe	Trypoxylini	
subfamily	Dinetinae	
subfamily	Mellininae	
subfamily	Pemphredoninae	
tribe	Entomosericini	
tribe	Pemphredonini	
tribe	Psenini	
subfamily	Philanthinae	
tribe	Cercerini	
tribe	Philanthini	
tribe	Pseudoscoliini	
family	Heterogynaidae	
family	Sphecidae	
tribe	Ammophilini	
tribe	Sceliphrini	
tribe	Sphecini	
superfamily	Ceraphronoidea	
family	Ceraphronidae	
family	Megaspilidae	
superfamily	Chalcidoidea	
family	Agaonidae	
subfamily	Agaoninae	
subfamily	Epichrysomallinae	
subfamily	Sycoryctinae	
family	Aphelinidae	
subfamily	Aphelininae	
subfamily	Azotinae	
subfamily	Calesinae	
subfamily	Coccophaginae	
subfamily	Eretmocerinae	
subfamily	Eriaporinae	
family	Chalcididae	
subfamily	Chalcidinae	
subfamily	Dirhininae	
subfamily	Epitraninae	
subfamily	Haltichellinae	
family	Encyrtidae	
subfamily	Encyrtinae	
subfamily	Tetracneminae	
family	Eucharitidae	
subfamily	Eucharitinae	
family	Eulophidae	
subfamily	Entedoninae	
subfamily	Euderinae	
subfamily	Eulophinae	
subfamily	Tetrastichinae	
family	Eupelmidae	
subfamily	Calosotinae	
subfamily	Eupelminae	
subfamily	Neanastatinae	
family	Eurytomidae	
subfamily	Eurytominae	
subfamily	Rileyinae	
family	Leucospidae	
family	Mymaridae	
family	Ormyridae	
family	Perilampidae	
subfamily	Chrysolampinae	
subfamily	Perilampinae	
subfamily	Philomidinae	
family	Pteromalidae	
subfamily	Asaphinae	
subfamily	Ceinae	
subfamily	Cerocephalinae	
subfamily	Cleonyminae	
subfamily	Colotrechninae	
subfamily	Cratominae	
subfamily	Diparinae	
subfamily	Eunotinae	
subfamily	Herbertiinae	
subfamily	Macromesinae	
subfamily	Miscogasterinae	
subfamily	Neodiparinae	
subfamily	Ormocerinae	
subfamily	Panstenoninae	
subfamily	Pireninae	
subfamily	Pteromalinae	
subfamily	Spalangiinae	
family	Signiphoridae	
family	Tetracampidae	
subfamily	Platynocheilinae	
subfamily	Tetracampinae	
family	Torymidae	
subfamily	Megastigminae	
subfamily	Toryminae	
family	Trichogrammatidae	
superfamily	Chrysidoidea	
family	Bethylidae	
family	Chrysididae	
subfamily	Chrysidinae	
tribe	Chrysidini	
tribe	Elampini	
tribe	Parnopini	
subfamily	Cleptinae	
family	Dryinidae	
family	Embolemidae	
family	Sclerogibbidae	
superfamily	Cynipoidea	
family	Cynipidae	
tribe	Aylacini	
tribe	Cynipini	
tribe	Diplolepidini	
tribe	Pediaspini	
tribe	Synergini	
family	Figitidae	
subfamily	Anacharitinae	
subfamily	Aspicerinae	
subfamily	Charipinae	
tribe	Alloxystini	
tribe	Charipini	
subfamily	Eucoilinae	
subfamily	Figitinae	
subfamily	Parnipinae	
family	Ibaliidae	
superfamily	Evanioidea	
family	Aulacidae	
family	Evaniidae	
family	Gasteruptiidae	
subfamily	Gasteruptiinae	
superfamily	Mymarommatoidea	
family	Mymarommatidae	
superfamily	Platygastroidea	
family	Platygastridae	
family	Scelionidae	
superfamily	Proctotrupoidea	
family	Diapriidae	
family	Heloridae	
family	Proctotrupidae	
family	Vanhorniidae	
superfamily	Stephanoidea	
family	Stephanidae	
subfamily	Stephaninae	
superfamily	Trigonaloidea	
family	Trigonalidae	
subfamily	Trigonalyinae	
superfamily	Vespoidea	
family	Bradynobaenidae	
family	Formicidae	
subfamily	Aenictinae	
subfamily	Dolichoderinae	
subfamily	Dorylinae	
subfamily	Formicinae	
subfamily	Leptanillinae	
subfamily	Myrmicinae	
subfamily	Ponerinae	
family	Mutillidae	
subfamily	Mutillinae	
subfamily	Myrmillinae	
subfamily	Myrmosinae	
subfamily	Pseudophotopsidinae	
subfamily	Sphaeropthalminae	
subfamily	Ticoplinae	
family	Pompilidae	
subfamily	Ceropalinae	
subfamily	Pepsinae	
tribe	Ageniellini	
tribe	Pepsini	
subfamily	Pompilinae	
tribe	Aporini	
tribe	Homonotini	
tribe	Pompilini	
tribe	Psammoderini	
family	Sapygidae	
family	Scoliidae	
subfamily	Proscoliinae	
subfamily	Scoliinae	
tribe	Campsomerini	
tribe	Scoliini	
family	Tiphiidae	
subfamily	Methochinae	
subfamily	Myzininae	
subfamily	Tiphiinae	
family	Vespidae	
subfamily	Eumeninae	
subfamily	Masarinae	
subfamily	Vespinae	

## Temporal coverage

**Living time period:** Currently living (extant).

### Notes

Currently living animals in stable populations, largely excluding (1) rare / irregular immigrants, intruder or invader species, (2) accidental or deliberate releases of exotic (pet)species, (3) domesticated animals, (4) foreign species imported and released for bio-control or (5) foreign species largely confined to hothouses.

## Usage rights

### Use license

Open Data Commons Attribution License

### IP rights notes

*Fauna Europaea* data are licensed under CC BY SA version 4.0. The property rights of experts over their data is covered under the SMEBD conditions. For more IPR details see: http://www.faunaeur.org/copyright.php.

## Data resources

### Data package title

Fauna Europaea - Hymenoptera - Apocrita

### Resource link

http://www.faunaeur.org/Data_papers/FaEu_Hymenoptera-Apocrita_2.6.2.zip

### Alternative identifiers

http://www.faunaeur.org/experts.php?id=662

### Number of data sets

2

### Data set 1.

#### Data set name

Fauna Europaea - Hymenoptera - Apocrita version 2.6.2 - species

#### Data format

CSV

#### Number of columns

25

#### Character set

UTF-8

#### Download URL

http://www.faunaeur.org/Data_papers/FaEu_Hymenoptera-Apocrita_2.6.2.zip

#### Description

**Data set 1.** 

Column label	Column description
datasetName	The name identifying the data set from which the record was derived (http://rs.tdwg.org/dwc/terms/datasetName).
version	Release version of data set.
versionIssued	Issue data of data set version.
rights	Information about rights held in and over the resource (http://purl.org/dc/terms/rights).
rightsHolder	A person or organization owning or managing rights over the resource (http://purl.org/dc/terms/rightsHolder).
accessRights	Information about who can access the resource or an indication of its security status (http://purl.org/dc/terms/accessRights).
taxonID	An identifier for the set of taxon information (http://rs.tdwg.org/dwc/terms/taxonID)
parentNameUsageID	An identifier for the name usage of the direct parent taxon (in a classification) of the most specific element of the scientificName (http://rs.tdwg.org/dwc/terms/parentNameUsageID).
scientificName	The full scientific name, with authorship and date information if known (http://rs.tdwg.org/dwc/terms/scientificName).
acceptedNameUsage	The full name, with authorship and date information if known, of the currently valid (zoological) taxon (http://rs.tdwg.org/dwc/terms/acceptedNameUsage).
originalNameUsage	The original combination (genus and species group names), as firstly established under the rules of the associated nomenclaturalCode (http://rs.tdwg.org/dwc/terms/originalNameUsage).
family	The full scientific name of the family in which the taxon is classified (http://rs.tdwg.org/dwc/terms/family).
familyNameId	An identifier for the family name.
genus	The full scientific name of the genus in which the taxon is classified (http://rs.tdwg.org/dwc/terms/genus).
subgenus	The full scientific name of the subgenus in which the taxon is classified. Values include the genus to avoid homonym confusion (http://rs.tdwg.org/dwc/terms/subgenus).
specificEpithet	The name of the first or species epithet of the scientificName (http://rs.tdwg.org/dwc/terms/specificEpithet).
infraspecificEpithet	The name of the lowest or terminal infraspecific epithet of the scientificName, excluding any rank designation (http://rs.tdwg.org/dwc/terms/infraspecificEpithet).
taxonRank	The taxonomic rank of the most specific name in the scientificName (http://rs.tdwg.org/dwc/terms/infraspecificEpithet).
scientificNameAuthorship	The authorship information for the scientificName formatted according to the conventions of the applicable nomenclaturalCode (http://rs.tdwg.org/dwc/terms/scientificNameAuthorship).
authorName	Author name information
namePublishedInYear	The four-digit year in which the scientificName was published (http://rs.tdwg.org/dwc/terms/namePublishedInYear).
Brackets	Annotation if authorship should be put between parentheses.
nomenclaturalCode	The nomenclatural code under which the scientificName is constructed (http://rs.tdwg.org/dwc/terms/nomenclaturalCode).
taxonomicStatus	The status of the use of the scientificName as a label for a taxon (http://rs.tdwg.org/dwc/terms/taxonomicStatus).
resourceDescription	An account of the resource, including a data-paper DOI (http://purl.org/dc/terms/description)

### Data set 2.

#### Data set name

Fauna Europaea - Hymenoptera - Apocrita version 2.6.2 - hierarchy

#### Data format

CSV

#### Number of columns

12

#### Character set

UTF-8

#### Download URL

http://www.faunaeur.org/Data_papers/FaEu_Hymenoptera-Apocrita_2.6.2.zip

#### Description

**Data set 2.** 

Column label	Column description
datasetName	The name identifying the data set from which the record was derived (http://rs.tdwg.org/dwc/terms/datasetName).
version	Release version of data set.
versionIssued	Issue data of data set version.
rights	Information about rights held in and over the resource (http://purl.org/dc/terms/rights).
rightsHolder	A person or organization owning or managing rights over the resource (http://purl.org/dc/terms/rightsHolder).
accessRights	Information about who can access the resource or an indication of its security status (http://purl.org/dc/terms/accessRights).
taxonName	The full scientific name of the higher-level taxon
scientificNameAuthorship	The authorship information for the scientificName formatted according to the conventions of the applicable nomenclaturalCode (http://rs.tdwg.org/dwc/terms/scientificNameAuthorship).
taxonRank	The taxonomic rank of the most specific name in the scientificName (http://rs.tdwg.org/dwc/terms/infraspecificEpithet).
taxonID	An identifier for the set of taxon information (http://rs.tdwg.org/dwc/terms/taxonID)
parentNameUsageID	An identifier for the name usage of the direct parent taxon (in a classification) of the most specific element of the scientificName (http://rs.tdwg.org/dwc/terms/parentNameUsageID).
resourceDescription	An account of the resource, including a data-paper DOI (http://purl.org/dc/terms/description)

## Figures and Tables

**Figure 4.**
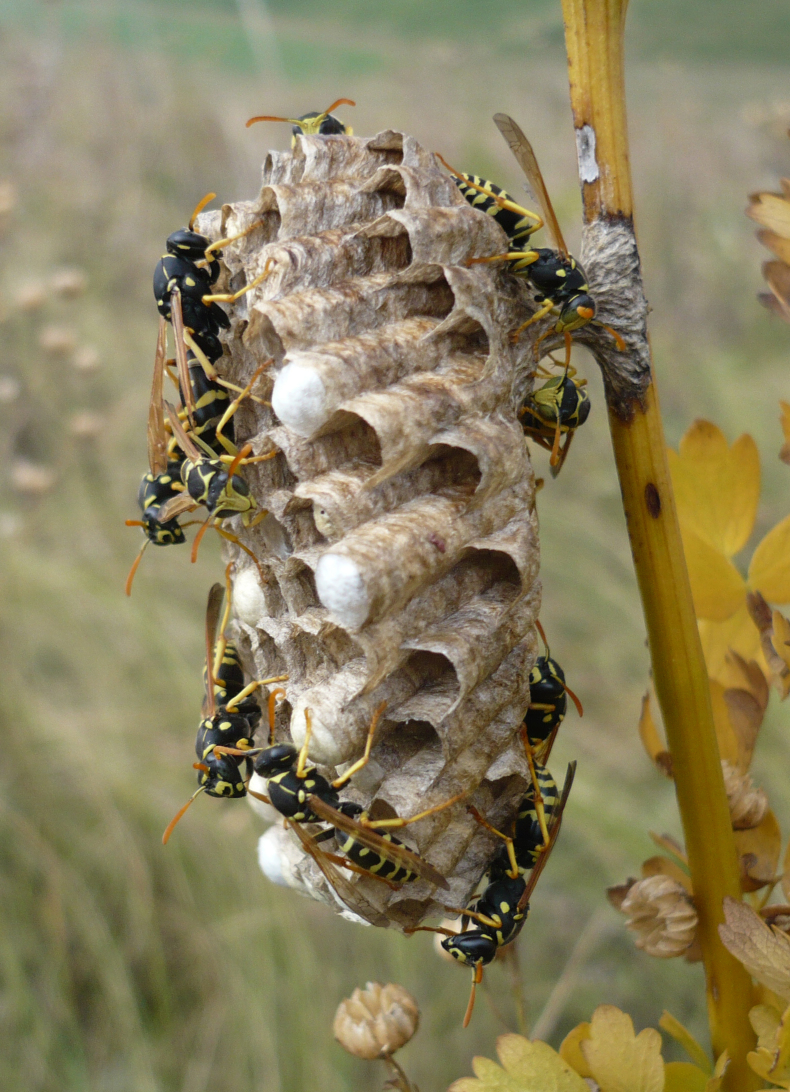
*Polistes* sp. (Vespoidea: Vespidae) nest.

**Figure 5.**
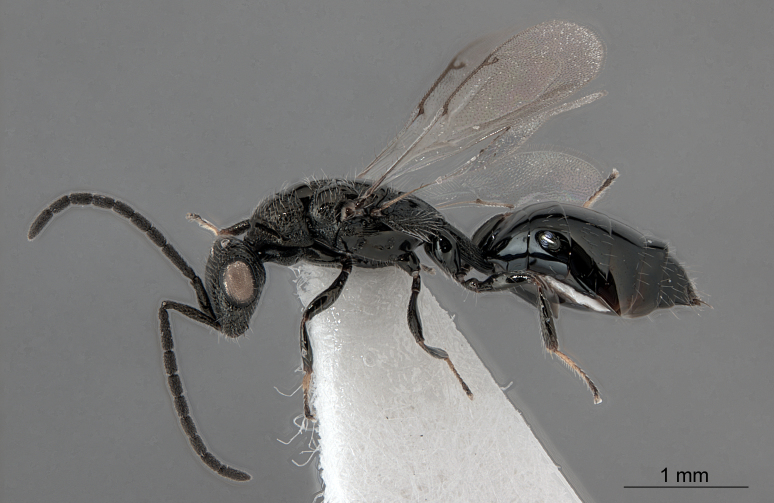
*Spalangia* sp. (Chalcidoidea: Pteromalidae).

**Figure 6.**
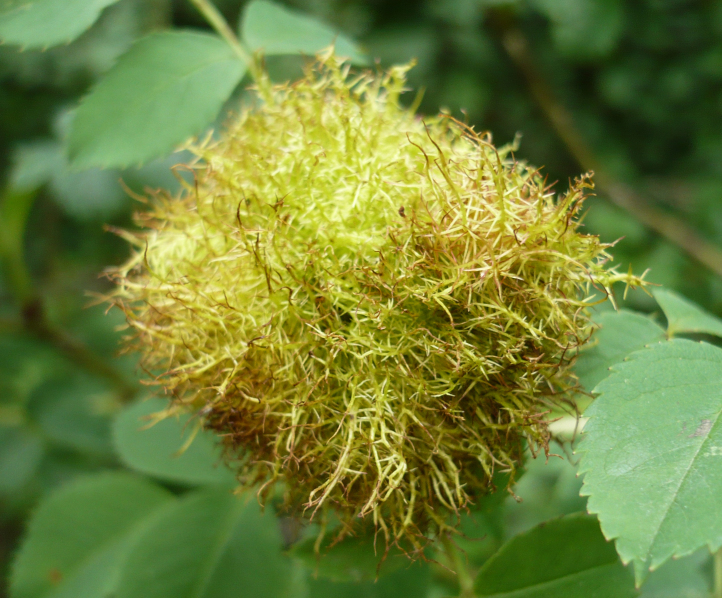
*Diplolepis* sp. (Cynipoidea: Cynipidae) gall on Rosa.

**Figure 7.**
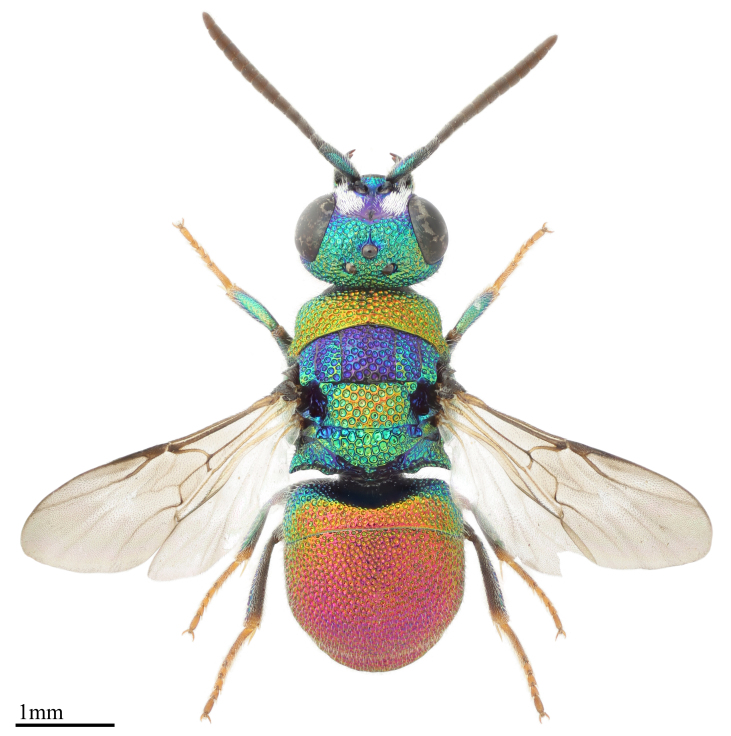
*Hedychridium vachali* Mercet, 1915 male from Spain (Chrysidoidea: Chrysididae). Author: Alexander Berg (courteously by chrysis.net).

**Table 1.** Responsible specialists per family in Hymenoptera - Apocrita (excluding Ichneumonoidea). The actual numbers of databased species are given per family. For most families also an indication of the actual number of known/described species (showing a potential information gap) is given plus an estimate of the total number of existing species (i.e., described/known plus undescribed/undiscovered) for Europe. * At present, Fauna Europaea lists 135 accepted chrysidid subspecies. We estimate that at least 50 subspecies could be considered as valid species, and future molecular analysis will prove it as in the case of the *Chrysis ignita* group (Soon et al. 2014). Based on recent unpublished findings, northern African species have been collected in the Iberian peninsula and S Italy, whereas Transcaucasian species are expected in Eastern Europe. Lastly, fifty-five European species and subspecies have been described after the publication of the world checklist of the Chrysididae by Kimsey & Bohart (Kimsey and Bohart 1990) and we expect around 50 species to be described in the next future, based on the material examined in different collections.

TAXONOMY		EUROPE		
FAMILY	SPECIALIST(S)	DATABASED SPECIES (Fauna Europaea)	TOTAL DESCRIBED SPECIES (information-gap)	TOTAL ESTIMATED SPECIES (knowledge-gap)
Agaonidae	Mircea-Dan Mitroiu	5	5	
Ampulicidae	Yvan Barbier	5	5	
Aphelinidae	Andrew Polaszek	193		
Apidae	Andrew Polaszek	2066		
Aulacidae	Michael Madl	11		
Bethylidae	Andrew Polaszek	226		
Bradynobaenidae	Guido Pagliano	5		
Ceraphronidae	Andrew Polaszek	102		
Chalcididae	Lucian Fusu	93	99	
Chrysididae	Oliver Niehuis / Paolo Rosa & Villu Soon	483	486	550-600 *
Crabronidae	Yvan Barbier	664	664	
Cynipidae	Fredrik Ronquist & Mattias Forshage	339	365	
Diapriidae	Norman Johnson	781		
Dryinidae	Massimo Olmi	107	114	
Embolemidae	Massimo Olmi	4	5	
Encyrtidae	Lucian Fusu	769	769	
Eucharitidae	Mircea-Dan Mitroiu	15	15	
Eulophidae	Mircea-Dan Mitroiu	1193	1193	
Eupelmidae	Lucian Fusu	105	118	
Eurytomidae	Mircea-Dan Mitroiu	352	353	
Evaniidae	Michael Madl	5		
Figitidae	Fredrik Ronquist & Mattias Forshage	425	440	
Formicidae	Alexander Radchenko	637		
Gasteruptiidae	Michael Madl	30		
Heloridae	Norman Johnson	4		
Heterogynaidae	Yvan Barbier	1	1	
Ibaliidae	Fredrik Ronquist & Mattias Forshage	3	3	
Leucospidae	Lucian Fusu	8	8	
Megaspilidae	Andrew Polaszek	140		
Mutillidae	Aleksandar Cetkovic & Guido Nonveiller	154	154	
Mymaridae	Lucian Fusu	457	457	
Mymarommatidae	Lucian Fusu	1	1	
Ormyridae	Mircea-Dan Mitroiu	25	25	
Perilampidae	Mircea-Dan Mitroiu	67	67	
Platygastridae	Norman Johnson	518		
Pompilidae	Raymond Wahis	284		
Proctotrupidae	Norman Johnson	59		
Pteromalidae	Mircea-Dan Mitroiu	1389	1391	
Sapygidae	Josef Gusenleitner	10	10	
Scelionidae	Norman Johnson	587		
Sclerogibbidae	Massimo Olmi	5	5	
Scoliidae	Till Osten	22		
Signiphoridae	Lucian Fusu	10	11	
Sphecidae	Yvan Barbier	58	58	
Stephanidae	Michael Madl	2		
Tetracampidae	Mircea-Dan Mitroiu	11	11	
Tiphiidae	Mario Boni Bartalucci	37		
Torymidae	Petr Jansta	326		
Trichogrammatidae	Lucian Fusu	147	147	
Trigonalidae	Michael Madl	1		
Vanhorniidae	Norman Johnson	1		
Vespidae	Josef Gusenleitner	269	271	

**Table 2.** Fauna Europaea Hymenoptera Apocrita excluding Ichneumonoidea expertise network status and changes.

FAMILY NAME	EXPERTS VERSIONS 1 & 2 (current)	Comment
Ampulicidae, Crabronidae, Heterogynaidae, Sphecidae	Yvan Barbier	active
Tiphiidae	Mario Boni Bartalucci	active
Mutillidae	Aleksandar Cetkovic & Guido Nonveiller	active (Guido Nonveiller deceased)
Chalcididae, Encyrtidae, Eupelmidae, Leucospidae, Mymaridae, Mymarommatidae, Signiphoridae, Trichogrammatidae	John Noyes (v.1), Lucian Fusu	active
Sapygidae, Vespidae	Josef Gusenleitner	active
Torymidae	John Noyes (v.1), Petr Jansta	active
Diapriidae, Heloridae, Platygastridae, Proctotrupidae, Scelionidae, Vanhorniidae	Norman Johnson	active
Aulacidae, Evaniidae, Gasteruptiidae, Stephanidae, Trigonalyidae	Michael Madl	active
Agaonidae, Eucharitidae, Eulophidae, Eurytomidae, Ormyridae, Perilampidae, Pteromalidae, Tetracampidae	John Noyes (v.1), Mircea-Dan Mitroiu	active
Dryinidae, Embolemidae, Sclerogibbidae	Massimo Olmi	active
Scoliidae	Till Osten (Ohl 2013)	deceased
Bradynobaenidae	Guido Pagliano	active
Aphelinidae, Apidae, Bethylidae, Ceraphronidae, Megaspilidae	John Noyes (Aphelinidae in v.1), Andrew Polaszek	active
Formicidae	Alexander Radchenko	active
Cynipidae, Figitidae, Ibaliidae	Fredrik Ronquist, Mattias Forshage	active
Chrysididae	Oliver Niehuis (v.1), Paolo Rosa & Villu Soon	active
Pompilidae	Raymond Wahis	active
